# Data of sperm-entry inability in *Drosophila melanogaster* ovarian follicles that are depleted of s36 chorionic protein

**DOI:** 10.1016/j.dib.2017.03.052

**Published:** 2017-04-08

**Authors:** Athanassios D. Velentzas, Panagiotis D. Velentzas, Stamatia Katarachia, Vassiliki E. Mpakou, Issidora S. Papassideri, Dimitrios J. Stravopodis

**Affiliations:** Section of Cell Biology and Biophysics, Department of Biology, School of Science, National and Kapodistrian University of Athens (NKUA), Athens, Greece

**Keywords:** Chorion, *Drosophila*, Egg, Follicle, Oogenesis, Ovary, RNAi, s36, Sperm

## Abstract

This paper presents data associated with the research article entitled “Targeted downregulation of s36 protein unearths its cardinal role in chorion biogenesis and architecture during *Drosophila melanogaster* oogenesis” [1]. *Drosophila* chorion is produced by epithelial follicle cells and one of its functional serving role is egg fertilization through the micropyle, a specialized narrow channel at the anterior tip of the egg [2]. Sperm entry during fertilization is necessary for the egg to complete meiosis [3]. *D. melanogaster* flies being characterized by severe downregulation of the s36 chorionic protein, specifically in the follicle-cell compartment of their ovary, appear with impaired fly fertility (Velentzas et al., 2016) [1]. In an effort to further investigate whether the observed infertility in the s36-targeted flies derives from a fertilization failure, such as the inability of sperm to pass through egg׳s micropyle, we mated females carrying s36-depleted ovaries with males expressing the GFP protein either in their sperm tails, or in both their sperm tails and sperm heads.

**Specification Table**

**Table:** 

Subject area	*Biology*
More specific subject area	*Cell and Developmental Biology*
Type of data	*Confocal Laser Scanning micrographs*
How data were acquired	*Using a Nikon Eclipse C1 Confocal Laser Scanning Microscope (CLSM)*
Data format	*Analyzed data*
Experimental factors	*Female virgin control and s36-targeted flies were mated with dj-GFP or protamineB-eGFP; dj-GFP males. The deposited eggs were collected every one hour and observed under a Nikon CLSM*
Experimental features	*Comparison of successful fertilization levels between laid s36-depleted ovarian follicles and control ones*
Data source location	
Data accessibility	*All data are included in this article*

## Value of data

• Insemination and not sperm entry into mature follicles seems responsible for the activation of ovulation process in *D. melanogaster*: new prospects for control of oogenesis by sperm microenvironment.
• Flies carrying s36-depleted ovaries may serve as a primary model system for deciphering the sperm-regulated ovulation and egg-deposition rhythms in *D. melanogaster*, through the use of spermatozoa with various genetic backgrounds.
• Imaging and quantification of *D. melanogaster* fertilization via employment of transgenic -fluorescent- spermatozoa technology most likely provide a useful and valuable platform for the assessment of, other than s36, major chorionic-components’ contribution to follicles’ competence for efficient fecundity.

## 1 Data

**Fig. 1:**
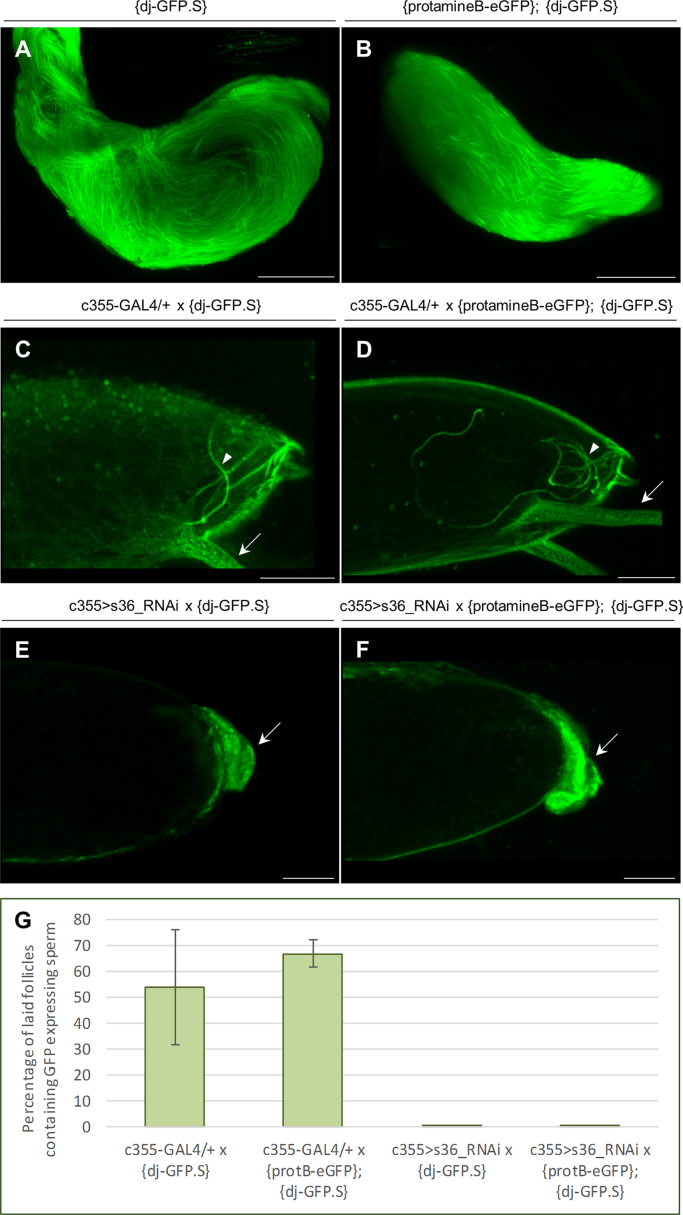
Fertilization inability of s36-depleted fly follicles results from sperm-entry failure. CLSM images of spermatozoa inside testes, expressing (A) the don juan (tail-specific) or (B) both the don juan and protamineB (head-specific) GFP-conjugated protein markers. CLSM images of laid fertilized eggs, as demonstrated by the GFP-tagged sperm inside each cytoplasm, after crossing control (c355-GAL4/+) female flies to (C) dj-GFP or (D) protamineB-eGFP; dj-GFP transgene-carrying males. Representative CLSM images of laid follicles, with no GFP-tagged sperm detected in any respective cytoplasm, having been derived from s36-targeted (c355>s36_RNAi) female flies after their mating with (E) dj-GFP or (F) protamineB-eGFP; dj-GFP transgene-containing males. (G) Graphic presentation of the percentage (%) of fertilized eggs, as indicated by the entry of fluorescent sperms through ovarian-follicles’ respective micropyles, for each one of the genetic backgrounds described above. Arrowheads point spermatozoa and arrows indicate dorsal appendages. Scale bars: 50 μm.

In order to examine *Drosophila melanogaster* sperm׳s ability to penetrate ovarian egg׳s micropyle [2] and enter into oocyte׳s cytoplasm of the s36-downregulated follicles, we mated s36-targeted virgin female flies with males expressing either the don juan-GFP fusion protein (dj-GFP), or both the dj-GFP and Mst35Bb/ProtamineB-eGFP proteins (Fig. 1A and B). The *Drosophila* don juan (dj) protein is expressed along the axoneme of each sperm tail [ 3–4], while protamineB is specifically localized in sperm heads [5]. To validate sperm׳s GFP-mediated fluorescence in the transgenic male flies, their testes expressing either the dj-GFP (Fig. 1A) or both the dj-GFP and protamineB-eGFP proteins (Fig. 1B) were visualized under a CLSM, clearly revealing bright green staining patterns for both spermatozoa populations examined.

More than half in number of the freshly-laid eggs (*n*=90) obtained from control (c355-GAL4/+) female flies after they have been crossed to males expressing dj-GFP (Fig. 1C and G) proved to be successfully fertilized, with GFP-tagged sperm being readily detected in their cytoplasm. Similarly, a 67% mean value of laid eggs (*n*=105), derived from control female flies mated with protamineB-eGFP; dj-GFP transgene-carrying males, were also presented with GFP-tagged sperm (see, its coiled shape within the anterior region of the herein shown representative follicle) inside each fertilized egg׳s cytoplasm (Fig. 1D and G). In contrast, GFP-tagged sperm could not be detected inside the cytoplasm of the freshly-laid s36-depleted eggs produced by female flies that have been inseminated either by dj-GFP (*n*=110; Fig. 1E and G) or by dj-GFP and protamineB-eGFP transgene-containing males (*n*=120; Fig. 1F and G). Interestingly, insemination (introduction of semen into the female animal), and not sperm penetration into the mature follicle, seems to represent a sufficient factor for triggering the ovulation process in *D. melanogaster*, since no statistically significant difference in the egg-deposition capacity could be observed between control and s36-targeted flies.

## 2 Experimental design, materials and methods

### 2.1 *Drosophila melanogaster* strain stocks and maintenance

For this study, the following *D. melanogaster* transgenic fly strains were used: P{w[+mW.hs]=GawB}c355, w[1118] (BL: 3750), w[*]; P{w[+mC]=protamineB-eGFP}2/CyO; P{w[+mC]=dj-GFP.S}3/TM3, Sb[1] (BL: 58406) and w[*]; P{w[+mC]=dj-GFP.S}AS1/CyO (BL: 5417), all obtained from Bloomington *Drosophila* Stock Center (Indiana, USA), and UAS-s36_RNAi (Transformant ID: 14824), provided by Vienna *Drosophila* RNAi Center (Vienna, Austria). Fly stocks maintenance was performed as previously described [1].

### 2.2 *Drosophila melanogaster* mating, egg collection and CLSM imaging

Control (c355-GAL4/+) and s36-depleted (c355>s36_RNAi) virgin female flies (3–5 days) were mated overnight with either dj-GFP.S or protamineB-eGFP; dj-GFP.S male flies. Female flies were left to lay their eggs in standard apple-juice agar plates and the obtained eggs were being collected every one hour and immediately observed under a Nikon confocal laser scanning microscope (CLSM), model Digital Eclipse C1 (Nikon; Tokyo, Japan).
